# Surgical Antimicrobial Prophylaxis in Pediatric Patients Undergoing Plastic Surgery: A RAND/UCLA Appropriateness Method Consensus Study

**DOI:** 10.3390/antibiotics11040506

**Published:** 2022-04-11

**Authors:** Susanna Esposito, Rossella Sgarzani, Sonia Bianchini, Sara Monaco, Laura Nicoletti, Erika Rigotti, Marilia Di Pietro, Roberta Opri, Caterina Caminiti, Matilde Ciccia, Giorgio Conti, Daniele Donà, Mario Giuffré, Stefania La Grutta, Laura Lancella, Mario Lima, Andrea Lo Vecchio, Gloria Pelizzo, Giorgio Piacentini, Carlo Pietrasanta, Matteo Puntoni, Alessandro Simonini, Elisabetta Venturini, Annamaria Staiano, Nicola Principi, on behalf of the Peri-Operative Prophylaxis in Neonatal and Paediatric Age (POP-NeoPed) Study Group

**Affiliations:** 1 Pediatric Clinic, University Hospital, Department of Medicine and Surgery, University of Parma, 43126 Parma, Italy; bianchini.sonia@outlook.it (S.B.); s.monaco1410@gmail.com (S.M.); laura.nicoletti@studenti.unipr.it (L.N.); 2 Servizio di Chirurgia Plastica, Centro Grandi Ustionati, Ospedale M. Bufalini, AUSL Romagna, 47521 Cesena, Italy; rossella.sgarzani@auslromagna.it; 3 Pediatric Clinic, Azienda Ospedaliera Universitaria Integrata, 37134 Verona, Italy; erika.rigotti@aovr.veneto.it (E.R.); marilia.dipietro@studenti.univr.it (M.D.P.); roberta.opri@gmail.com (R.O.); giorgio.piacentini@univr.it (G.P.); 4 Research and Innovation Unit, University Hospital of Parma, 43126 Parma, Italy; ccaminiti@ao.pr.it (C.C.); mpuntoni@ao.pr.it (M.P.); 5 Neonatology and Neonatal Intensive Care Unit, Ospedale Maggiore, 40133 Bologna, Italy; matilde.ciccia@aosp.bo.it; 6 Pediatric ICU and Trauma Center, Fondazione Policlinico Universitario A. Gemelli IRCCS, 00165 Rome, Italy; giorgio.conti@unicatt.it; 7 Division of Paediatric Infectious Diseases, Department for Woman and Child Health, University of Padua, 35100 Padua, Italy; daniele.dona@unipd.it; 8 Department of Health Promotion, Mother and Child Care, Internal Medicine and Medical Specialties “G. D’Alessandro”, University of Palermo, 90141 Palermo, Italy; mario.giuffre@unipa.it; 9 Institute of Translational Pharmacology IFT, National Research Council, 90146 Palermo, Italy; stefania.lagrutta@cnr.it; 10 Paediatric and Infectious Disease Unit, Academic Department of Pediatrics, IRCCS Bambino Gesù Children’s Hospital, 00165 Rome, Italy; laura.lancella@opbg.net; 11 Pediatric Surgery, IRCCS Azienda Ospedaliera-Universitaria di Bologna, 40138 Bologna, Italy; mario.lima@unibo.it; 12 Department of Translational Medical Science, Section of Pediatrics, University of Naples “Federico II”, 80138 Naples, Italy; andrealovecchio@gmail.com (A.L.V.); staiano@unina.it (A.S.); 13 Pediatric Surgery Department, “Vittore Buzzi” Children’s Hospital, 20154 Milano, Italy; gloria.pelizzo@unimi.it; 14 Neonatal Intensive Care Unit, Fondazione IRCCS Ca’ Granda Ospedale Maggiore Policlinico, Department of Mother, Child and Infant, 20122 Milan, Italy; carlo.pietrasanta@unimi.it; 15 Pediatric Anesthesia and Intensive Care Unit, Salesi Children’s Hospital, 60123 Ancona, Italy; dr.simonini@gmail.com; 16 Pediatric Infectious Disease Unit, Meyer’s Children Hospital, 50139 Florence, Italy; elisabetta.venturini@meyer.it; 17 Università degli Studi di Milano, 20122 Milan, Italy; nicola.principi@unimi.it

**Keywords:** antibiotics, burn, pediatric infectious diseases, plastic surgery, surgical antibiotic prophylaxis, wound

## Abstract

For many years, it was clearly shown that surgical procedures might be associated with surgical site infection (SSI). Many scientific institutions prepared guidelines to use in surgery to reduce abuse and misuse of antibiotics. However, in the general guidelines for surgical antibiotic prophylaxis, plastic surgical procedures are not addressed or are only marginally discussed, and children were almost systematically excluded. The main aim of this Consensus document is to provide clinicians with recommendations on antimicrobial prophylaxis for pediatric patients undergoing plastic surgery. The following scenarios were considered: clean plastic surgery in elective procedures with an exclusive skin and subcutis involvement; clean-contaminated/contaminated plastic surgery in elective procedures with an exclusive skin and subcutis involvement; elective plastic surgery with use of local flaps; elective plastic surgery with the use of grafts; prolonged elective plastic surgery; acute burns; clean contused lacerated wounds without bone exposure; high-risk contused lacerated wounds or with bone exposure; contused lacerated wound involving the oral mucosa; plastic surgery following human bite; plastic surgery following animal bite; plastic surgery with tissue expander insertion. Our Consensus document shows that antimicrobial perioperative prophylaxis in pediatric patients undergoing plastic surgery is recommended in selected cases. While waiting the results of further pediatric studies, the application of uniform and shared protocols in these procedures will improve surgical practice, with a reduction in SSIs and consequent rationalization of resources and costs, as well as limiting the phenomenon of antimicrobial resistance.

## 1. Introduction

For many years, it was clearly shown that surgical procedures might be associated with surgical site infection (SSI). However, the risk of SSI could significantly vary according to the type and duration of the surgical procedure, the patient’s characteristics, the antibiotic’s choice, and the administration schedule. This should have led to defining which surgical procedures can benefit from antibiotic administration and from which antibiotics, and which administration schedule can offer the best protection with the highest safety and tolerability and the lowest cost for the health system. Unfortunately, this did not happen for a long time, and antibiotics were almost systematically prescribed to all the patients undergoing a surgical procedure [1,2,3]. Abuse and misuse of antibiotics were associated with a prolonged hospital stay, increased drug-related adverse events, and reduced efficacy of the antimicrobial agents. Relevance was the evidence that antibiotic abuse and misuse were the leading causes of the increase in the emergence of resistance to the most frequently prescribed antibiotics of the commonest bacterial pathogens, with dramatic impact on the incidence of antibiotic therapy failures [4,5].

The awareness of the importance of rational use of antibiotics has led to planning several studies to better define when and how prophylaxis is needed. Many scientific institutions used the results to prepare guidelines which could be used in surgery. However, some branches of surgery have been more deeply studied than others. In some cases, it is now well defined which patients can benefit from antibiotic prophylaxis and how this should be performed; for others, the present knowledge remains poor, and the risk of antibiotic misuse remains virtually unchanged. Plastic surgical procedures are absolutely among the least studied, and this explains why in the general guidelines for surgical antibiotic prophylaxis, plastic surgical procedures are not addressed or are only marginally discussed [6]. Moreover, contrarily to what has been produced for other surgical procedures, children were almost systematically excluded in the few studies regarding plastic surgery. The main aim of this Consensus document is to provide clinicians with a series of recommendations on antimicrobial prophylaxis for pediatric patients undergoing plastic surgery.

## 2. Methods

### 2.1. RAND/UCLA Appropriateness Method

This Consensus document was realized using the Research and Development Corporation (RAND) and the University of California, Los Angeles (UCLA) appropriateness method. The RAND/UCLA method consists of the appropriateness evaluation of diagnostic and therapeutic procedures with suboptimal scientific evidence by a panel of experts [7]. According to the RAND method, a procedure is defined as “appropriate” if the expected benefits outweigh the expected negative consequences, with a wide margin that justifies it, regardless of the costs. In contrast, a procedure whose expected risks outweigh the expected benefits is considered “inappropriate”. According to the RAND definition, experts who make an appropriateness/inappropriateness judgment must consider the clinical benefits and not be influenced by economic considerations. Therefore, appropriateness is used to evaluate the risk/benefit ratio of a list of diagnostic, management and therapeutic procedures [8]. For a heterogeneous topic such as surgical antimicrobial prophylaxis on which randomized controlled trials in pediatrics are lacking, the application of methods aiming to increase the homogeneity of behaviors by neonatologists, infectious diseases specialists, pediatric surgeons, and anesthetists appeared useful and appropriate. For this reason, the RAND/UCLA approach was chosen instead of the Grading of Recommendations Assessment, Development and Evaluation (GRADE) methodology. Through the RAND method, the participants discussed different clinical scenarios and elaborated statements based on the literature and their clinical experience. The group of experts did not consider it appropriate to combine the GRADE method with the RAND/UCLA approach because the absence of randomized studies represents a bias in defining the strength of the recommendations and in representing a consensus reached for real life.

### 2.2. Recruitment of Panelists

A multidisciplinary group of experts belonging to the main Italian scientific societies dealing with anti-infective therapy of children was selected. The following Scientific Societies were involved: Italian Society of Pediatrics (SIP), Italian Society of Neonatology (SIN), Italian Society of Pediatric Infectious Diseases (SITIP), Italian Society of Infectious and Tropical Diseases (SIMIT), Italian Society of Pediatric Surgery (SICP), Italian Society of Microbiology (SIM), Italian Society of Pharmacology (SIF), Italian Society of Anesthesia and Neonatal and Pediatric Resuscitation (SARNEPI), Italian Society of Childhood Respiratory Diseases (SIMRI) and Italian Society of Plastic Surgery (SICPRE). The panel of experts was made up of 52 physicians with at least a 5-year experience: pediatricians (*n* = 20), neonatologists (*n* = 6), infectious diseases specialists (*n* = 5), pediatric surgeons (*n* = 5), anesthetists (*n* = 8), pharmacologist (*n* = 5) and microbiologists (*n* = 3). Participants were identified among the main experts in the field by the President and the Board of Directors of each participating Scientific Society.

### 2.3. Generation of Scenarios

Initially, a literature search was performed with a selection of documents, including randomized studies, systematic reviews of the literature, meta-analyses and guidelines on perioperative prophylaxis for the prevention of SSI during plastic surgery. The literature search was carried out on the PubMed database, with a choice of articles in English published from 2000 until 2020. The key search terms were: “antimicrobial prophylaxis” OR “antibiotic prophylaxis” AND “plastic surgery” OR “burn” OR “wound” OR “bite” AND “neonate” OR “newborn” OR “paediatric” OR “pediatric” OR “children” OR “adolescent”. All identified publications were considered and are reported in the references of this manuscript. Subsequently, using the Patient/Problem/Population–Intervention–Comparison/Control/Comparator–Outcome (PICO) model (i.e., defining a clinical question in terms of the specific patient problem), a questionnaire was created on perioperative prophylaxis during plastic surgery in neonatal and pediatric patients, which was divided into 12 clinical scenarios. Before administration, it was tested twice with a one-week interval to a convenience sample of 4 pediatricians, 2 neonatologists, one infectious diseases specialist, one pediatric surgeon, one anesthetist, one pharmacologist and one microbiologist. All 4 pediatricians were experts in antimicrobial stewardship. Then, 26 out of 52 experts were selected by the Scientific Societies for answering, and the questionnaire was administered to 11 pediatricians, 3 neonatologists, 2 infectious diseases specialists, 3 pediatric surgeons, 4 anesthetists, 2 pharmacologists, and one microbiologist. Selected experts were those that agreed to participate actively in the discussion on the Consensus document.

### 2.4. Two-Round Consensus Process

Based on the scenarios, the questionnaire was submitted to experts on the “REDCap” electronic data capture tools hosted at the University Hospital of Parma [9]. REDCap (Research Electronic Data Capture) is a secure, web-based software platform designed to support data capture for research studies, providing: (1) an intuitive interface for validated data capture; (2) audit trails for tracking data manipulation and export procedures; (3) automated export procedures for seamless data downloads to common statistical packages; and (4) procedures for data integration and interoperability with external sources. Each question included the clinical scenario and possible answers on whether SAP was recommended for the scenario and, in case of its recommendation, a list with all the antibiotics available on the European Union market such that the expert could select the antibiotics that he/she considered as first choice. The selected bibliographic material was made available to all panel members, who were instructed on how to fill out the questionnaire. The experts answered anonymously to the questionnaire, and their judgement was expressed on a 1-9 scale, where “1” was considered definitely inappropriate, “5” was considered uncertain, and “9” was considered appropriate. Intermediate values corresponded to different modulations of the judgement of inappropriateness (“2” and “3”), uncertainty (from “4” to “6”) and appropriateness (“7” and “8”). In evaluating each indication, each expert referred both to their own experience and clinical judgement and to the available scientific evidence. A free space was provided for any annotation or comment.

The first round of the questionnaire was blinded to the other panel members. Multiple participation was not permitted by the platform, which also guaranteed the confidentiality and anonymity of the answers. The results of the survey were discussed in a collegial meeting, with all 26 experts who answered the questionnaire, to reach agreements and reduce eventual disagreements [8]. Clarifications, adaptations, and refinements of the indications and appropriateness ratings were made. A total of 12 recommendations were developed. Participants were asked to approve the recommendations in a second round during the following four weeks.

## 3. Results

Table 1 shows the main application areas of plastic surgery in pediatric population.

### 3.1. SCENARIO #1—Antimicrobial Prophylaxis in Pediatric Patients Undergoing Clean Plastic Surgery in Elective Procedures with Exclusive Skin and Subcutis Involvement

In this group, patients requiring surgical procedures involving skin and subcutis in different body parts and breast surgery are included, and a surgical procedure does not imply the use of flaps or grafts. Unfortunately, most of the studies that have evaluated antibiotic prophylaxis in these patients have significant methodological limitations leading to serious risk of bias and low-quality results. However, apart from breast surgery for which perioperative antibiotic prophylaxis was shown effective, all the other types of clean plastic surgery studies have shown that the risk of SSI development is low and that antibiotics do not modify this risk. A meta-analysis of all the studies (randomized and nonrandomized controlled trials; 67 studies met the inclusion criteria) published before June 2015 carried out by the American Association of Plastic Surgeons [10] revealed that in clean hand and limb surgery, risk of infection was 0.6% in patients with prophylaxis and 0.9% in those without (odds ratio (OR) 0.81, 95% confidence interval (CI) 0.40–1.66, *p* = 0.56); in clean head and neck surgery 2.4% vs. 3.7% (OR 0.49, 95% CI 0.19–1.23, *p* = 0.13) and in clean skin surgery 1.9% vs. 5.2% (OR 0.54, 95% CI 021–1.42, *p* = 0.21). As the total number of enrolled patients was generally low and antibiotic regimes ranged from a single preoperative dose to prolonged postsurgical administration, conclusions regarding potential difference according to the schedule could not be evaluated. Although these findings had a low level of evidence, several scientific institutions concluded against the use of antibiotic prophylaxis in clean plastic surgery with exclusive skin and subcutis involvement. Breast surgery was considered an exception [10,11,12]. Data collected in children are few. They seem to indicate that young patients can have SSIs in about 1–4% of cases [13,14,15,16]. However, our panel of experts also concluded against antibiotic prophylaxis in this procedure.

Recommendation 1. Antibiotic surgical prophylaxis is not recommended in pediatric patients undergoing clean plastic surgery in elective procedures with exclusive skin and subcutis involvement.

### 3.2. SCENARIO #2—Antimicrobial Prophylaxis in Pediatric Patients Undergoing Clean-Contaminated/Contaminated Plastic Surgery in Elective Procedures with Exclusive Skin and Subcutis Involvement

In some cases, elective plastic surgery procedures involving skin and subcutis (which do not imply the use of flaps or grafts) can be considered clean-contaminated or contaminated as the operative wound enters the respiratory or the alimentary tract due to fistulas, or surgery is performed on inflamed tissue. In these conditions, the risk of SSI development can be theoretically significant, with *Staphylococcus aureus*, *Staphylococcus epidermidis*, *Streptococcus pyogenes* and *Pseudomonas aeruginosa* as the most common bacterial pathogens [17]. Clinical trials did not clarify this problem. Efficacy of antibiotic prophylaxis in adults varied significantly according to the type of surgery and characteristics of the studies. Moreover, to complicate the final evaluation, in some studies, a certain number of patients with risk factors such as the use of prostheses or skin flaps was considered. In hand and limb surgery, prophylaxis was found effective in randomized controlled trials. SSIs were diagnosed in 5.1% of patients who received antibiotic prophylaxis and in 7.7% of controls (OR 0.54, 95% CI 0.30–0.96, *p* = 0.04). On the contrary, when nonrandomized controlled studies were added to the evaluation, the difference between treated and untreated patients was not more significant (4.9% vs. 5.7%, *p* = 0.76, 95% CI 0.49–1.17, *p* = 0.13) [18,19,20,21,22,23,24,25,26,27]. In head and neck surgery, a relevant reduction of SSI incidence was shown when only randomized (16.4% vs. 41.9%; OR 0.23, 95% CI 0.11–0.46, *p* < 0.0001) and randomized plus nonrandomized studies (12.2% vs. 25.7%; 95% CI 0.12–0.54; *p* < 0.0001) were analyzed [28,29,30,31,32,33,34,35], leading to the conclusion that relevance of antibiotic prophylaxis could not be established [10]. Despite these conflicting results and the poor quality of clinical trials, most scientific societies agree to recommend the use of antibiotic prophylaxis in adults undergoing all these types of plastic surgery [6,16]. Generally, cefazolin i.v. (2 g) is recommended preoperatively and 4 h after procedure. Alternatively, some authors mention ampicillin–sulbactam.

In a pediatric setting, the evidence is poor. However, results of some studies seem to indicate that the level of wound contamination and the duration of the procedure play a major role in conditioning risk of SSI [13,14]. Suggested molecules for children are the same as recommended for adults.

Recommendation 2. Antibiotic prophylaxis with a pre-operative dose of cefazoline 30 mg/kg (max 2 g) i.v. is recommended in pediatric patients undergoing elective clean-contaminated/contaminated plastic surgery with exclusive skin and subcutis involvement.

### 3.3. SCENARIO #3—Antimicrobial Prophylaxis in Pediatric Patients Undergoing Elective Plastic Surgery with the Use of Local Flaps

The results of studies in patients undergoing elective plastic surgery with the use of flaps generally indicate that these subjects are at increased risk of SSIs versus patients with simple clean surgery [6].

A multicenter study carried out in 3491 adult patients undergoing reconstructive procedures, with 90.2% using flaps, has documented an incidence of SSIs of about 4.3%, compared with 1.9% for simple clean surgery [36]. In a prospective study by Dixon et al., enrolling 2434 adult patients undergoing 5091 dermatological surgical procedures, an infection rate of 0.54% for simple excisions and 2.94% for flaps was documented [37]. However, a more accurate analysis of enrolled cases reported that the risk of SSIs was significant only for the use of flaps in positions below the knee, as in this case, the incidence of SSIs rose to above 5%. This explains why some authors have recommended that the use of perioperative antibiotic prophylaxis in patients undergoing skin flap was reserved only to selected cases such as those with flaps below the knee or when they involve other at-risk sites (i.e., nose, ear, armpit, lip, groin) [11,38]. In the Wright et al. guidelines, the use of amoxicillin is indicated for procedures involving the oral or nasal mucosa or cefazolin in those involving the leg [11]. The pediatric field is even more uncertain due to the paucity of high-quality studies.

Recommendation 3. In pediatric patients undergoing elective plastic surgery with the use of flaps, perioperative antibiotic prophylaxis is not recommended. In cases involving at-risk sites (i.e., leg below the knee, nose, ear, armpit, lip, groin), cefazolin 30 mg/kg (max 2 g) i.v. within the 30 min before surgery is recommended.

### 3.4. SCENARIO #4—Antimicrobial Prophylaxis in Pediatric Patients Undergoing Elective Plastic Surgery with the Use of Grafts

The use of grafts leads to a higher risk of SSIs than clean surgery. A multicenter study including 3491 dermatologic surgical procedures showed that incidence of SSIs was 1.6% after clean procedures with exclusive skin and subcutis involvement and 4.3% after skin graft reconstruction [36]. An even higher rate (8.7%) was reported in a prospective study enrolling 2424 patients [37]. This explains why the use of prophylaxis is generally recommended [11,37,38]. However, recommendations regarding drug of choice vary significantly. Amoxicillin–clavulanic acid is the combination of choice when the procedure involves the oral or nasal mucosa, and cefazolin is preferred in the other cases [11,16,38]. Antibiotics are given pre-operatively and in the first 24 h after the procedure. The indications in the pediatric field are even more uncertain, due to the limited number of high-quality studies.

Recommendation 4. In pediatric patients undergoing elective plastic surgery with the use of grafts, antibiotic prophylaxis with amoxicillin–clavulanic acid (50 mg/kg as amoxicillin) oral or i.v. or ampicillin–sulbactam (50 mg/kg as ampicillin) i.v. when the procedure involves the oral or nasal mucosa and cefazolin 30 mg/kg (max 2 g) i.v. in the other cases within the 30 min before surgery and in the first 24 h after the procedure are recommended.

### 3.5. SCENARIO #5—Antimicrobial Prophylaxis in Pediatric Patients Undergoing any Type of Prolonged Elective Plastic Surgery

Several prospective and retrospective studies have identified a greater risk of SSIs in interventions lasting more than 2 h, even in the pediatric setting [39]. Surgery duration was shown to be an important predictor of surgical site infection in a retrospective study of 58498 patients undergoing different types of surgery [40]. Similarly, Garibaldi et al. found in a study of 1852 patients that procedures lasting longer than 2 h tripled the infection rate [41]. In the dermatology field, a prospective study of 1100 patients undergoing elective reconstructive or cosmetic procedures indicated that antibiotic prophylaxis should be performed if the operation lasted more than 3 h [42]. A meta-analysis by Zhang et al. showed that antibiotic prophylaxis was able to reduce the infection rate in clean plastic surgery with potential risk factors, such as the duration of the surgical procedure [43]. In a pediatric setting, the most significant risk factors appeared to be the level of wound contamination and the duration of surgery [13,14,44]. The molecule most used in the literature for antibiotic prophylaxis is cefazolin for its cost, efficacy, the safety of use and duration of action [43].

Recommendation 5. In pediatric patients undergoing any type of elective plastic surgery lasting more than 2 h, cefazolin 30 mg/kg (max 2 g) i.v. within the 30 min before surgery is recommended, repeatable in case of surgery lasting more than 4 h.

### 3.6. SCENARIO #6—Antimicrobial Prophylaxis in Pediatric Patients Undergoing Plastic Surgery for Acute Burns

Use of antibiotics to prevent infection development is not generally recommended in burned adults and children. This is because prophylactic systemic antimicrobials have never been shown to reduce infections or sepsis and can, on the contrary, increase the incidence of resistant organisms in the patient [45,46,47,48,49]. Ergun et al. compared the use of antibiotic prophylaxis in 77 pediatric burn patients and did not demonstrate a higher incidence of infection in the placebo group [45]. Similarly, Mulgrev et al. compared the use of antibiotic prophylaxis in 1250 burn patients over a 16-year follow-up period, considering two groups: the control group, who received antibiotics when clinically necessary, and the group who received antibiotics as routine prophylaxis [47]. This study shows no statistical differences between the two groups, both in terms of morbidity and infectious complications [47].

Moreover, WHO guidelines, based on the available evidence, conclude not supporting the role of systemic antibiotic prophylaxis in the management of pediatric burns [49]. However, in burned adults requiring reconstructive surgical procedure after extensive burns, perioperative antibiotic prophylaxis can be considered. Surgery of acute burn patients consists of escharotomy and reconstruction with skin grafts, skin substitutes or flaps. Although cefazolin seems to be a narrow-spectrum antibiotic if extensive burns are considered, the literature showed that cefazolin given preoperatively and in the first 24 h after procedure is the first choice [6]. No specific studies regarding children are available.

Recommendation 6. In pediatric patients undergoing reconstructive plastic surgery after burns, antibiotic prophylaxis is not recommended. In case of extensive burns and when the surgery includes the insertion of flaps or a graft, cefazolin 30 mg/kg (max 2 g) i.v. given within the 30 min before surgery and every 4 h during the first 24 h after the procedure is recommended. Before proceeding with skin grafting, a preoperative culture swab is recommended.

### 3.7. SCENARIO #7—Antimicrobial Prophylaxis in Pediatric Patients Undergoing Plastic Surgery following Clean Contused Lacerated Wounds without Bone Exposure

Adult patients with clean contused lacerated wounds without bone exposure undergoing plastic surgery are at low risk of SSIs [50]. Moreover, antibiotic prophylaxis does not reduce this risk and may even increase it [51,52]. A retrospective study enrolling 330 patients revealed that less than 10% of these kinds of wounds was contaminated by bacteria, and only 1.2% of subjects later developed wound infections [53]. A double-blind randomized controlled multicenter study comparing the rate of SSIs between patients receiving cefalexin or clindamycin or placebo showed that only 1% developed infections without difference between groups [54]. In a meta-analysis of seven studies, wound infection rates in controls ranged from 1.1% to 12% (average of 6%). Patients treated with antibiotics (cefazolin, cephalexin, flucloxacillin) had a slightly higher risk of infection than untreated controls [50].

According to the Academy of Emergency Medicine and Care (AcEMC) and the World Society of Emergency Surgery (WSES), antibiotic prophylaxis is unnecessary in immunocompetent patients with low-risk wounds [55]. No sufficient data have been collected in children to draw definitive conclusions. Regardless of antibiotic prophylaxis, the need for anti-tetanus prophylaxis should be assessed [55].

Recommendation 7. In pediatric patients undergoing plastic surgery following clean contused lacerated wounds without bone exposure, perioperative antibiotic prophylaxis is not recommended.

### 3.8. SCENARIO #8—Antimicrobial Prophylaxis in Pediatric Patients Undergoing Plastic Surgery following High-Risk Contused Lacerated Wounds or with Bone Exposure

Despite limited evidence, several reviews recommended antibiotic prophylaxis in the case of a contaminated wound [55,56,57,58], including in pediatrics especially for high-risk injuries [59]. Cardany et al. showed that crush injuries were at higher risk of SSIs, presumably due to the presence of devitalized tissue [60], and Moran et al. indicated antibiotic prophylaxis as necessary in these cases [58]. Of special interest are plantar puncture wounds or exposed fractures because of the high risk of complications such as osteomyelitis [61]. An extensive literature review [62] as well as the meta-analysis by Aryan et al. [10] concluded for the need of antibiotic prophylaxis in severely contaminated wounds. Traumatic wounds involving bone or poorly vascularized areas are also considered to be at high-risk for SSIs [57]. Many authors have indicated that antibiotic prophylaxis is necessary in case of contused lacerated wounds with involvement of cartilage and tendons, of joints or in the presence of fractures [51,57,58].

According to the AcEMC and the WSES, antibiotic prophylaxis is indicated for contaminated contused lacerated wounds or with bone involvement [55]. In both cases, cefazolin is the most often recommended antimicrobial agent [55]. Although a first-generation cephalosporin with or without an aminoglycoside is recommended for most patients, penicillin should be added to provide additional anaerobic coverage in severely contaminated wounds [63]. However, in neonates and children undergoing emergency surgery for the reduction of an exposed grade III fracture or traumatic amputation, our study group recommended peri-operative prophylaxis with cefazolin within 30 min before surgery, except in cases where broad-spectrum antibiotic therapy is already in progress [64].

Concerning the duration of prophylaxis, there is a relative agreement between various authors, who indicate that antibiotic coverage is required ranging from 72 h to 5 days following the trauma [51]. Once again, data in children are few and do not allow to draw firm conclusions [16]. Regardless of antibiotic prophylaxis, the need for anti-tetanus prophylaxis should be assessed [55].

Recommendation 8. In pediatric patients undergoing plastic surgery following high-risk contused lacerated wounds or bone exposure, cefazolin 30 mg/kg (max 2 g) i.v. given within 30 min before surgery and every 4 h for the first 72 h after the procedure is recommended.

### 3.9. SCENARIO #9—Antimicrobial Prophylaxis in Pediatric Patients Undergoing Plastic Surgery following Contused Lacerated Wound Involving the Oral Mucosa

Some authors indicated that antibiotic prophylaxis is necessary in case of involvement of the oral mucosa [58], while Mark et al., in a review considering older studies, concluded that prophylactic oral antibiotics played an inconclusive role in the treatment of intraoral wounds and recommended their use based on the physician’s clinical judgment, waiting for larger clinical trials [65]. Altieri et al. studied the benefits of 3 days of penicillin prophylaxis in a randomized control study on 100 intraoral lacerations managed in a pediatric emergency room [66]. The overall infection rate was 6.4%, with no statistically significant differences between the control (8.5%) and penicillin (4.4%) groups. Although this study had a limited number of patients enrolled, it concluded that routine antibiotic prophylaxis was not justified for simple intraoral lacerations in children [67]. A 2007 review suggested that antimicrobial prophylaxis was useful for intraoral full-thickness lacerations in adults, while it did not find sufficient evidence to make the same recommendations in children [51]. Another extensive literature review recommended antimicrobial prophylaxis use in full thickness lesions but did not identify sufficient evidence to make recommendations for tongue and intraoral lacerations [62]. Recent international expert opinions [67] and the AcEMC [55] recommend the use of antibiotic prophylaxis with amoxicillin–clavulanate as the molecule of choice. Regardless of antibiotic prophylaxis, the need for anti-tetanus prophylaxis should be assessed [55].

Recommendation 9. In pediatric patients undergoing plastic surgery following contused lacerated wounds involving the oral mucosa, antibiotic prophylaxis with oral amoxicillin–clavulanate (at a dose of 50 mg/kg as amoxicillin) or with ampicillin–sulbactam (at a dose of 50 mg/kg as ampicillin) i.v. given within the 30 min before surgery is recommended.

### 3.10. SCENARIO #10—Antimicrobial Prophylaxis in Pediatric Patients Undergoing Plastic Surgery following Human Bite

Some authors indicate, in the case of plastic surgery following a human bite, to perform antibiotic prophylaxis [68]; others suggest it based on the location: only at the level of the head [69], hands [70], or feet and cartilage areas [71]. Bula-Rudas et al., in an extensive pediatric review, recommend prophylaxis only in the case of comorbidities, high-risk or puncture wounds, bites that occurred more than 6 h before, and extensive soft tissues trauma [72]. Overall, most guidelines recommend its use [73].

The therapeutic alternatives proposed in the literature are many, and ideally, the first dose should be administered parenterally to obtain adequate tissue levels as quickly as possible [73]. In children, ampicillin–sulbactam is the drug of choice for the initial intravenous dose, whereas amoxicillin–clavulanate is the recommended oral drug [73]. Some authors suggest combining metronidazole with oral amoxicillin–clavulanate therapy [74,75], while others in severe cases recommend adding vancomycin to ampicillin–sulbactam [72].

Recommendation 10. In pediatric patients undergoing plastic surgery following a human bite, antibiotic prophylaxis with ampicillin–sulbactam (at a dose of 50 mg/kg as ampicillin) i.v. or with oral amoxicillin–clavulanate (at a dose of 50 mg/kg as amoxicillin) given within the 30 min before surgery and every 8 h during the first 24 h is recommended.

### 3.11. SCENARIO #11—Antimicrobial Prophylaxis in Pediatric Patients Undergoing Plastic Surgery following Animal Bite

The routine use of antibiotic prophylaxis for dog bite wounds is controversial because some recent studies have shown that infection rates have been low, ranging from 2% to 5% [76,77]. Conversely, feline bites are rarer than dog bites, but with infection rates of up to 50% because the wounds are puncture-like, and are therefore deeper and at increased risk of infection [78].

Several authors suggested prophylaxis in high-risk dog bites (i.e., in case of edema or crush injuries, devitalized tissue or full-thickness wounds involving tendons, ligaments and joints, at the level of the face, hands, feet and of the genital area) and in all types of cat bites [51,79,80,81].

Looke and Dendle, in a systematic review on the effectiveness and safety of antibiotic prophylaxis in human bites and non-human bites, recommended prophylactic antibiotics only in the case of risk sites, even for a cat bite [81]. The same indication is provided by the Infectious Disease Society of America, in whose guidelines prophylaxis is recommended only in patients or sites at risk (i.e., immune suppression, asplenia, liver disease, edema of the affected area, injuries to the hand or face or lesions that may have penetrated the periosteum or joint capsule) [73].

Data are lacking on bites due to animals different from dogs and cats [80].

The Infectious Disease Society of America stated that when antibiotic prophylaxis is administered, ideally, the first dose should be given parenterally to achieve effective tissue levels as quickly as possible [73]. Therapeutic alternatives are numerous in the literature: ampicillin–sulbactam is the drug of choice for the starting dose in children; amoxicillin–clavulanate is the recommended oral drug [73,82].

Zangari et al., in a review about the clinical records for 127 pediatric patients affected by dog-related injuries, and Jenkins at al., in a randomized controlled trial, suggest associating metronidazole with oral therapy with amoxicillin–clavulanate [74,75]. Bula-Rudas et al., in an extensive pediatric review, recommend adding vancomycin to ampicillin–sulbactam [72].

Regarding the duration of therapy, an extensive retrospective study on 5000 adult and pediatric patients showed that antibiotic prophylaxis performed for less than 48 h proved to be of little value with infection rates of around 75% [73]. The authors therefore considered that the optimal duration could be 5 days [73]. In various reviews, the authors have provided an indication to continue prophylaxis for 3–5 days [51,74] or for 5–7 days [14]. The Infectious Disease Society of America provides 5 days of antibiotic prophylactic therapy as required duration [73].

Regardless of antibiotic prophylaxis, the need for anti-tetanus and anti-rabies prophylaxis should be assessed [80].

Recommendation 11. In pediatric patients undergoing plastic surgery following animal bite, antibiotic prophylaxis with ampicillin–sulbactam (at a dose of 50 mg/kg as ampicillin) i.v. or with oral amoxicillin–clavulanate (at a dose of 50 mg/kg as amoxicillin) given within the 30 min before surgery and every 8 h for 5 days is recommended.

### 3.12. SCENARIO #12—Antimicrobial Prophylaxis in Pediatric Patients Undergoing Plastic Surgery with Tissue Expander Insertion

Tissue expansion is an important technique used in reconstruction of skin and soft-tissue defects, such as congenital nevi, burn and trauma scars, and genitourinary defects [83]. Due to the intrinsic presence of the tissue expansion, despite the application of meticulous surgical techniques and high sterility, these kinds of procedures have a high rate of complications (up to 40%), mainly in pediatric patients [83,84,85,86,87,88].

In an analysis of 264 pediatric operations involving tissue expander placement, Wang et al. identified specific risk factors for complications (reported in 16.5% of the cases, with a 10.8% premature removal of the implanted expanders): young age (0–6 years), type of surgery (i.e., for burn reconstructions), anatomical site (lower extremities and scalp), and number of expanders placed during a single operation [89]. In addition, Azadgoli’s study highlighted the importance of the number of expanders placed at the same operation as a risk factor for infection [89].

Although previous reports indicated a routine discharge on a course of oral antibiotic [89,90], in a review of 317 pediatric non-breast expander placement operations, Wang et al. observed that 3–7-day prolonged post-operative prophylactic antibiotics did not affect rates of postoperative infection, compared to a first-generation cephalosporin given only until maximum the first 24 h after surgery [91].

Similar findings were reported by the group of Phillips referring to breast reconstruction, for which the authors recommend a 24 h course of perioperative antibiotic [90].

Recommendation 12. In pediatric patients undergoing plastic surgery with tissue expander insertion, cefazolin 30 mg/kg (max 2 g) i.v. given within the 30 min before surgery is recommended.

## 4. Discussion

Widely shared clinical practice guidelines for antibiotic prophylaxis in patients undergoing plastic surgery are not presently available. This is because no definitive conclusions can be drawn from most of the studies that have focused on this problem, as many of them have several methodological issues. Moreover, in several studies, clinical conditions with different risks of SSIs are considered together with further difficulties in the evaluation of the risk. This explains why recommendations for antibiotic prophylaxis published by some scientific institutions differ significantly. The only aspect on which everyone agrees is the distinction between clean interventions and clean-contaminate/contaminated procedures, with prophylaxis not recommended in the former and with antibiotics considered potentially useful in the latter. However, especially for clean-contaminated infections, the classification of individual cases is not always uniform. Further limitation is represented by the fact that there is a lack of precise evaluations of the type of antibiotic most suitable for each individual circumstance and of the ways in which it should be administered.

The situation is even worse for interventions involving children because the studies that have enrolled pediatric subjects are limited or completely absent for the different clinical scenarios that may require plastic surgery. Waiting for a significant expansion of knowledge relating to antibiotic prophylaxis in plastic surgery in children, the latter can only follow the recommendations formulated for adults, at least those shared by all.

This Consensus document aimed to respond to issues that are still little addressed, with the ambition to fill current shortcomings. The specific scenarios developed are intended to guide the healthcare professional in practice to ensure a better and standardized management of the neonatal and pediatric patient. Table 2 summarizes the 12 recommendations for antibiotic prophylaxis in pediatric plastic surgery. Recommendations for allergic patients and for those with specific high-risk conditions are summarized in another article of our study group [92].

The strengths of this work are an updated literature review, the use of a rigorous analysis method (RAND/UCLA), the involvement of many exponents of the most important Italian scientific societies, and the specific consideration of age. The potential limitation of this study is the scarcity of data in the literature, which is partly overcome by the involvement of numerous and selected experts. The participants in the project came from different clinical contexts, i.e., they were pediatricians, neonatologists, infectious diseases specialists, pediatric surgeons, anesthetists, pharmacologists and microbiologists. Another limitation is that this was an opinion-based survey. Conversely, the lack of pediatric studies on the selected topics did not permit the use of the GRADE methodology, and the complexity of the topics required an online face-to-face meeting with all participants. The group of experts did not consider it appropriate to combine the GRADE method with the RAND/UCLA approach because the absence of randomized studies represents a bias in defining the strength of the recommendations and in representing a consensus reached for real life. The RAND/UCLA method did not permit to define a hierarchy of antibiotics administration, and not using the GRADE method may affect the quality of these recommendations. However, the findings obtained can establish the basis for educational interventions that aim to optimize the use of antibiotics.

## 5. Conclusions

**Figure 1:**
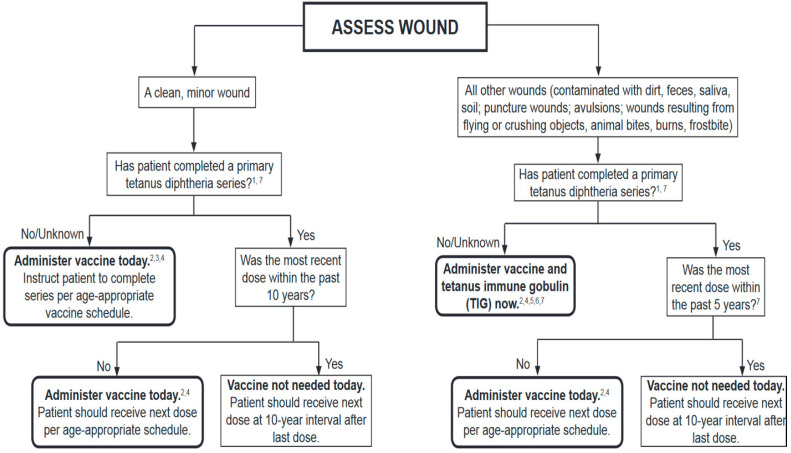
Summary guide to tetanus prophylaxis in routine wound management. ^1^ A primary series consists of a minimum of 3 doses of tetanus- and diphtheria-containing vaccine (DTaP/DTP/Tdap/DT/Td), ^2^ Age-appropriate vaccine: DTaP for infants and children 6 weeks up to 7 years of age; Tetanus–diphtheria (Td) toxoid for persons 7 through 9 years of age; Tdap for persons 11 through 18 years of age, ^3^ No vaccine or Tetanus Immune Globulin (TIG) is recommended for infants younger than 6 weeks of age with clean, minor wounds (no vaccine is licensed for infants younger than 6 weeks of age), ^4^ Tdap is preferred for persons 11 through 64 years of age if using Adacel or 10 years of age and older if using Boostrix who have never received Tdap. Td is preferred to tetanus toxoid (TT) for persons 7 through 9 years, 65 years and older, or who have received a Tdap previously. If TT is administered, adsorbed TT product is preferred to fluid TT. All DTaP/DTP/Tdap/DT/Td products contain adsorbed tetanus toxoid. ^5^ Give TIG 250 U IM for all ages. It can and should be given simultaneously with the tetanus-containing vaccine. ^6^ For infants younger than 6 weeks of age, TIG (without vaccine) is recommended for ‘’dirty” wounds (wounds other than clean, minor). ^7^ Persons who are HIV positive should receive TIG regardless of tetanus immunization history.

Our Consensus document shows that antimicrobial perioperative prophylaxis in pediatric patients undergoing plastic surgery is recommended in selected cases. This study, made possible by the multidisciplinary contribution of experts belonging to the most important Italian scientific societies, represents, in our opinion, the most up-to-date and comprehensive collection of recommendations relating to the behaviors to be undertaken in plastic surgery in pediatric age. While waiting for the results of further pediatric studies, the application of uniform and shared protocols in these procedures will improve surgical practice, with a reduction in SSIs and consequent rationalization of resources and costs, as well as limiting the phenomenon of antimicrobial resistance. In addition, as shown in Figure 1, tetanus prophylaxis for wound management must always be remembered [92].

## Figures and Tables

**Table 1:** Major plastic surgery procedures in neonatal and pediatric age.

1. Clean Elective Procedures without Flaps or Grafts
Congenital skin lesions or vascular lesions excision Otoplasty
**2. Clean-Contaminated/Contaminated Elective Procedures without Flaps or Grafts**
Cystic lesions excision Ingrown toenail correction Skin lesions of any kind with fistula to respiratory or alimentary tract
**3. Elective Procedures with Local Flaps**
Head and neck, hand and limb, urinary malformations (for example, cleft lip and palate, syndactyly) Scar contractures release—scar revision Chronic wounds (pressure sores)
**4. Elective Procedures with Grafts**
Skin grafts/bone grafts/nerve grafts/lipofilling Malformations (for example, bone graft in the alveolar process in complete cleft lip and palate) Scar contractures release—scar revision
**5. Prolonged Elective Procedures (More than 2 h)**
Complex malformations (for example, craniosynostosis, rare clefts) Oncologic surgery and reconstruction with free flaps
**6. Acute Burns**
Escarectomy and skin graft or flap
**7. Clean Contused Lacerated Wounds without Bone Exposure**
**8. High-Risk Contused Lacerated Wounds or with Bone Exposure**
**9. Contused Lacerated Wound Involving the oral Mucosa**
**10. Human Bite**
**11. Animal Bite**
**12. Elective Procedure with Skin Expander Insertion**
Congenital skin lesion (giant congenital nevus) Scar revision—excision

**Table 2:** Recommendation of antibiotic prophylaxis in pediatric plastic surgery.

Type of Plastic Surgical Procedure	Recommendation
Clean plastic surgery in elective procedures	Not recommended
Clean-contaminated/contaminated plastic surgery in elective procedures	Cefazoline 30 mg/kg (maximum dose 2 g) IV within 30 min before surgery
Elective plastic surgery with the use of flaps	Not recommended In cases involving at-risk sites (i.e., leg below the knee, nose, ear, armpit, lip, groin) cefazolin 30 mg/kg (maximum dose 2 g) IV within the 30 min before surgery
Elective plastic surgery with the use of graft	Amoxicillin–clavulanic acid (50 mg/kg as amoxicillin) oral or IV or ampicillin–sulbactam (50 mg/kg as ampicillin) IV when the procedure involves the oral or nasal mucosa. Cefazolin 30 mg/kg (maximum dose 2 g) IV in the other cases within the 30 min before surgery and in the first 24 h after the procedure
Prolonged elective plastic surgery (lasting more than 2 h)	Cefazolin 30 mg/kg (maximum dose 2 g) IV within the 30 min before surgery, repeatable in case of surgery lasting more than 4 h
Plastic surgery following acute burns	When the surgery includes insertion or flaps or graft, cefazolin 30 mg/kg (maximum dose 2 g) IV given within the 30 min before surgery and every 4 h during the first 24 h after the procedure
Plastic surgery following clean contused lacerated wounds without bone exposure	Not recommended.

## Data Availability

All the data are included in the manuscript.
